# Supplementary material for: Zingerone in the Flower of Passiflora maliformis Attracts an Australian Fruit Fly, Bactrocera jarvisi (Tryon)
Source: Molecules. 2020 Jun 22;25(12):2877. doi: 10.3390/molecules25122877 (PMC7355451; doi:10.3390/molecules25122877)
Supplement: Supplementary file 1 [file molecules-25-02877-s001.pdf]

molecules

Supplementary Materials

Zingerone in the Flower of *Passiflora Maliformis* Attracts an Australian Fruit Fly, *Bactrocera Jarvisi* (Tryon)

Soo Jean Park [1,*], Stefano G. De Faveri [2], Jodie Cheesman [2], Benjamin L. Hanssen [3], Donald N. S. Cameron [1], Ian M. Jamie [3] and Phillip W. Taylor [1]

[1] Applied BioSciences, Faculty of Science and Engineering, Macquarie University, Sydney, NSW 2109, Australia; donald.cameron@mq.edu.au (D.N.S.C.); phil.taylor@mq.edu.au (P.W.T.)
[2] Horticulture and Forestry Science, Queensland Department of Agriculture and Fisheries, Mareeba, QLD 4880, Australia; stefano.defaveri@daf.qld.gov.au (S.G.D.F.); jodie.cheesman@daf.qld.gov.au (J.C.)
[3] Department of Molecular Sciences, Faculty of Science and Engineering, Macquarie University, Sydney, NSW 2109, Australia; benjamin.hanssen@hdr.mq.edu.au (B.L.H.); ian.jamie@mq.edu.au (I.M.J.)
* Correspondence: soojean.park@mq.edu.au; +61-41-361-6107 or +61-29-850-1316

General Procedure

^{1}H and ^{13}C nuclear magnetic resonance (NMR) spectra were recorded using a Bruker Avance DPX 400 NMR spectrometer operating at 400 MHz for ^{1}H NMR, 101 MHz for ^{13}C NMR. $CDCl_3$ was used as the solvent for all NMR samples. ^{1}H NMR chemical shifts are reported in parts per million (δ) referenced to the proton signal of the deuterated solvent ($CDCl_3$: 7.26 ppm). ^{13}C NMR chemical shifts are reported in parts per million (δ) referenced to the carbon signal of the deuterated solvent ($CDCl_3$: 77.16 ppm). The following abbreviations are used to describe the NMR data – singlet (s), doublet (d), multiplet (m), and broad singlet (bs). Low resolution mass spectra were recorded on a Shimadzu GCMS-2010 using electron impact ionisation (EI) (70 eV). The progress of reaction was monitored with thin layer chromatography (TLC) and was performed using Merck TLC silica gel 60 F_{254} on aluminium sheets (0.2 mm) and visualised with ultraviolet light at 254 nm. Solvents were removed under reduced pressure using a Büchi Rotavapor R-200, Büchi V-500 vacuum pump, and Büchi B-490 heating bath set to a temperature of 40 °C. Drying following solvent removal was performed with an Alcatel Pascal 2005 SD high vacuum pump. Flash column chromatography was performed using Merck 60 silica gel (40–60 µm). All reagents/solvents were purchased from Sigma-Aldrich, Merck, or Alfa-Aesar and used without further purification.

Synthesis of Zingerol (4-(4-Hydroxy-3-Methoxyphenyl)-2-Butanol)

To a solution of zingerone (1.00 g, 5.15 mmol, 1.0 eq) in methanol (2.00 mL) was added a suspension of sodium borohydride (0.150 g, 3.97 mmol, 3.1 eq) in methanol (1.00 mL). The yellow suspension was stirred at room temperature for 90 minutes. Water (10.0 mL) was then added to the stirring yellow suspension. The product was extracted with ethyl acetate (3 × 10.0 mL) and the combined organic layers were washed with saturated brine solution (3 × 30.0 mL) and dried with $MgSO_4$. The solvent was removed under reduced pressure to yield the pure product as a very pale yellow oil (0.976 g, 4.97 mmol, 96.6%, R_f: 0.09 (3:1 (*v*/*v*) hexane:ethyl acetate)). ^{1}H NMR (400 MHz, $CDCl_3$): δ 1.23 (3 H, d, *J* = 6.2 Hz, CH(OH)C**H**$_3$), 1.75 (2 H, m, CH_2C**H**$_2$CH(OH)-), 2.65 (2 H, m, -CC**H**$_2$CH(OH)-), 3.83 (1 H, m, CH_2C**H**(OH)CH_3), 3.88 (3 H, s, OC**H**$_3$), 5.46 (1 H, bs, O**H**), 6.69 (2 H, m, **H**^{ar}), 6.83 (1 H, d, *J* = 7.7 Hz, **H**^{ar}) ppm. ^{13}C NMR (101 MHz, $CDCl_3$): δ 23.8, 32.0, 41.3, 56.0, 67.7, 111.1, 114.4, 121.0, 134.1, 143.8, 146.6 ppm. GCMS (EI) *m*/*z* (%) 196 (M^+, 35), 137 (M – C_3H_6OH, 100). Experimental spectral data were consistent with literature data[1].

NMR and Mass Spectral Data of Unknown Compounds

3-ethyl-2,3-dihydro-4*H*-pyran-4-one. ^{1}H NMR (400 MHz, $CDCl_3$): δ 0.89 (3 H, m, C**H**$_3$), 1.62 (2 H, m, C**H**$_2$), 2.78 (1 H, m, COC**H**(CH_2CH_3)CH_2), 4.28 (2 H, dd, *J* = 12.0 Hz, *J* = 4.6 Hz, OC**H**$_2$CH), 5.09 (1 H, m, CHC**H**CO), 7.37 (1H, m, OC**H**CH_2). ^{13}C NMR (101 MHz, $CDCl_3$), δ 12.2, 17.9, 27.3, 59.3, 71.9, 118.2, 172.2, 204.6.

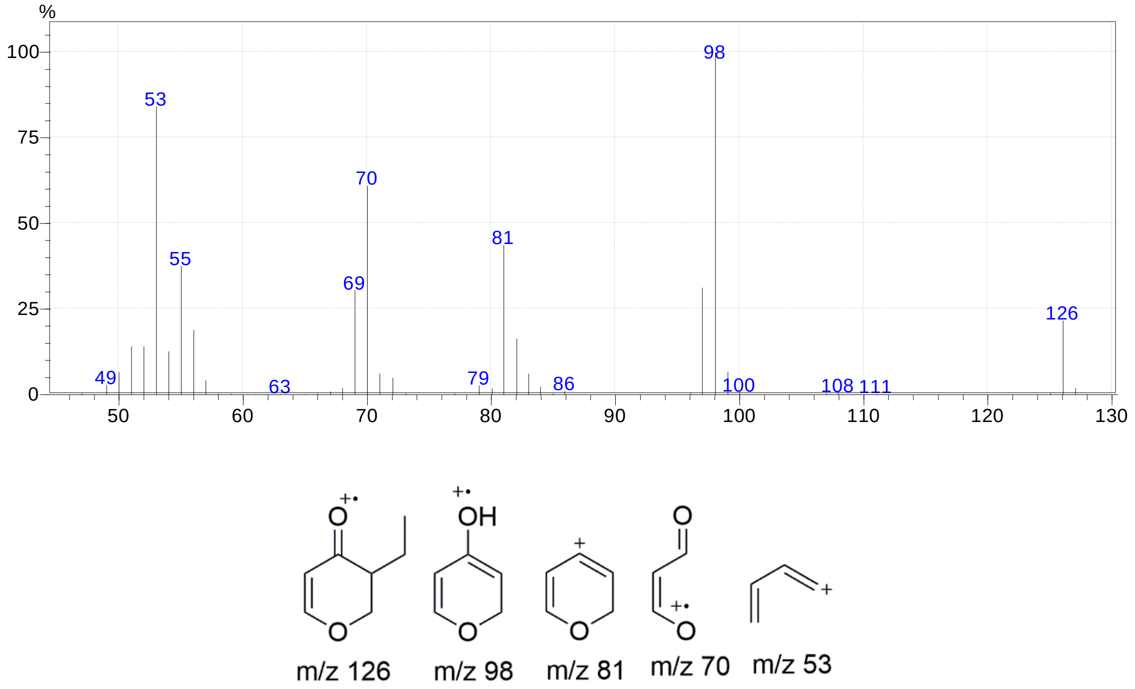

Figure S1. Mass spectrum and diagnostic ions of 3-ethyl-2,3-dihydro-4*H*-pyran-4-one.

1-(4-methyl-1,3-dioxolan-2-yl)pentan-3-one. ^{1}H NMR (400 MHz, $CDCl_3$): δ 1.03 (3 H, m, CH_2**CH_3**), 1.65 (3 H, m, CH**CH_3**), 1.85 (2 H, m, CH**CH_2**CH_2), 2.25 (2 H, m, CH_2CO**CH_2**CH_3, or **CH_2**CO$CH_2$$CH_3$), 2.35 (2 H, m, CH_2CO**CH_2**CH_3, or **CH_2**CO$CH_2$$CH_3$), 4.16 (1 H, dd, *J* = 11.9 Hz, *J* = 4.5 Hz, O**CH_2**CH), 4.28 (1 H, dd, *J* = 11.9 Hz, *J* = 4.5 Hz, O**CH_2**CH), 5.11 (1 H, m, O(CH_3)**CH**CH_2O), 5.36 (1 H, m, OC**H**O); ^{13}C NMR (101 MHz, $CDCl_3$), δ 12.0, 14.1, 27.3,34.1, 37.3, 39.7, 71.9, 77.2, 121.7, 211.1.

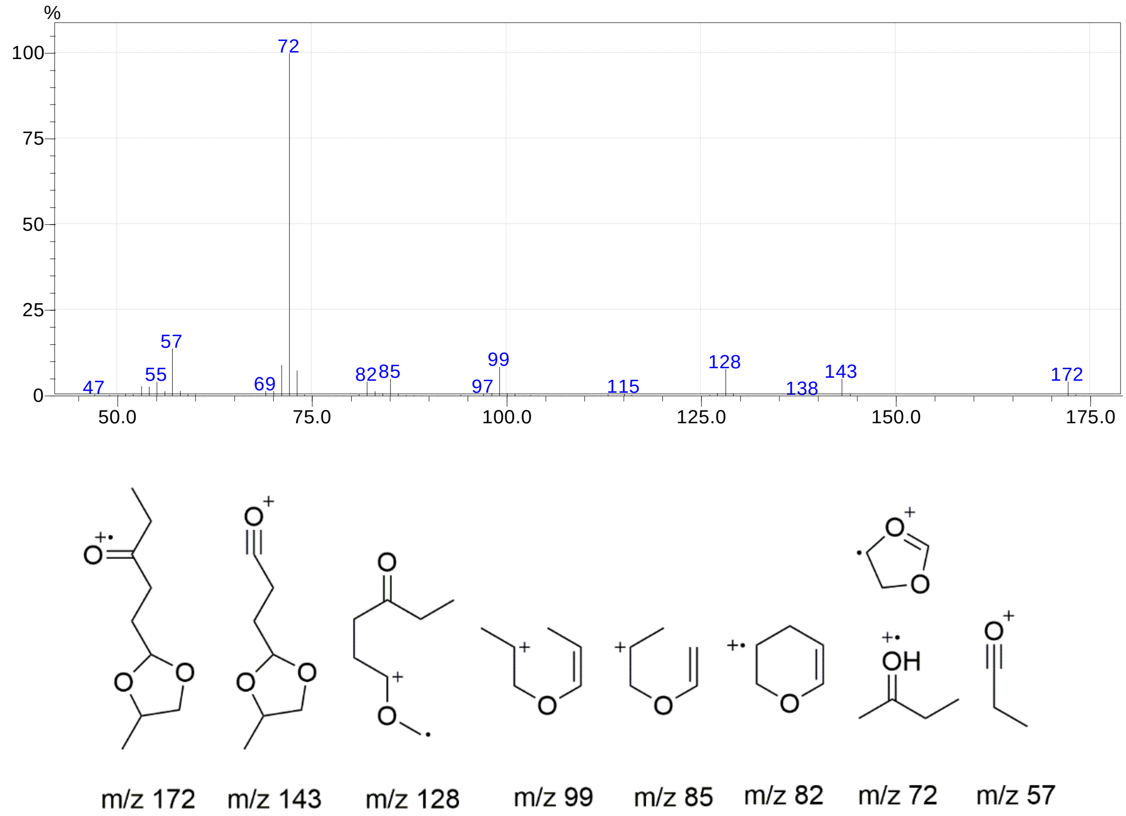

Figure S2. Mass spectrum and diagnostic ions of 1-(4-methyl-1,3-dioxolan-2-yl)pentan-3-one (RI 1227).

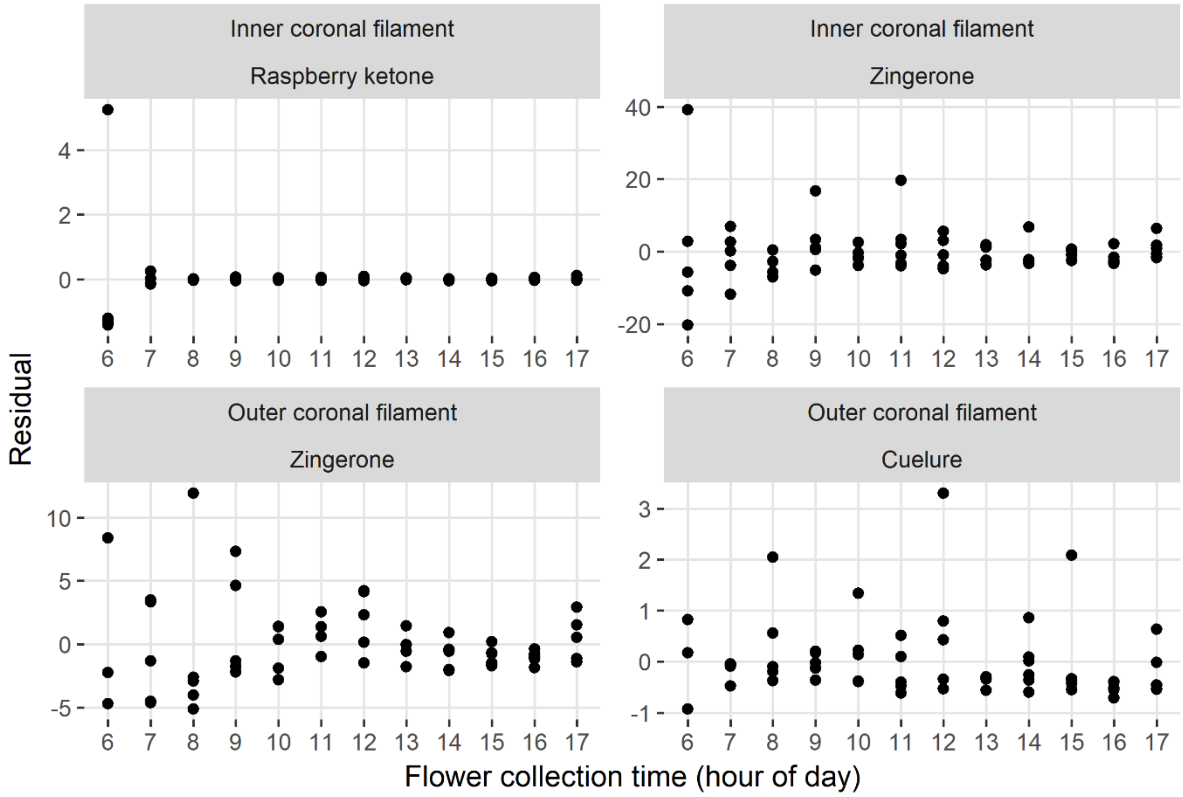

Figure S3. Residual plots of raspberry ketone in the inner coronal filament, zingerone in both filaments and cuelure in the outer coronal filament from exponential decay fitting.

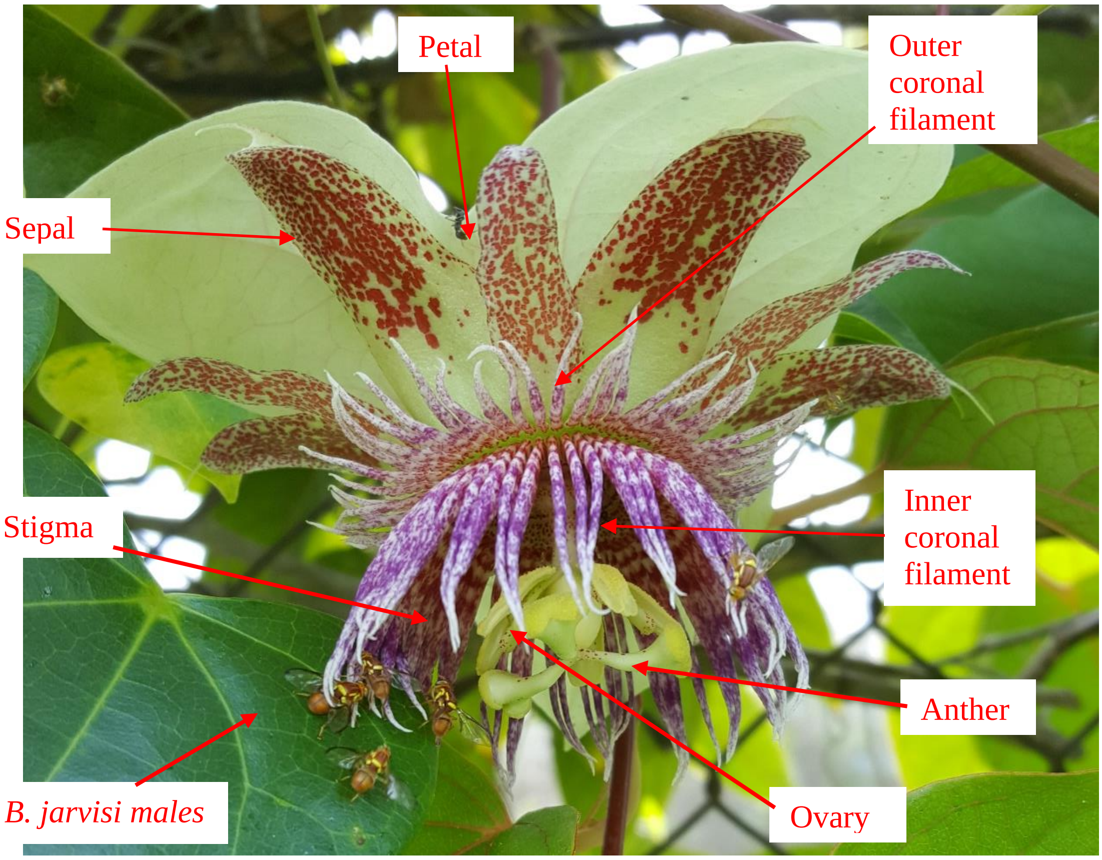

Figure S4. Flower of *P. maliformis* and *B. jarvisi* males visiting.

References

1. Agarwal, M.;Walia, S.;Dhingra, S., and Khambay, B.P.S. Insect growth inhibition, antifeedant and antifungal activity of compounds isolated/derived from *Zingiber officinale* Roscoe (ginger) rhizomes. *Pest Manage. Sci.*, **2001**. *57*, 289–300.
